# T-cell responses to SARS-CoV-2 vaccinations in adults with Down syndrome – a prospective cohort study

**DOI:** 10.1080/21645515.2025.2583416

**Published:** 2025-11-06

**Authors:** Lobke C. M. Hensen, Bianca M. M. Streng, Femke van Wijk, Stefan Nierkens, Rob S. van Binnendijk, Anne-Marie Buisman, Antonia M. W. Coppus, Corine H. Geurts van Kessel, Gert de Graaf, Fiona R. van der Klis, Regina Lamberts, Gestur Vidarsson, Tracy J. Ruckwardt, Esther de Vries, Rory D. de Vries, Michel E. Weijerman, Daniel M. Weinberger, Joanne G. Wildenbeest, Louis J. Bont, Eveline M. Delemarre

**Affiliations:** a Department of Paediatric Infectious Diseases and Immunology, Wilhelmina Children’s Hospital, University Medical Centre Utrecht, Utrecht, The Netherlands; b Center for Translational Immunology, University Medical Centre Utrecht, Utrecht, The Netherlands; c Princess Máxima Centre for Paediatric Oncology, Utrecht University, Utrecht, The Netherlands; d Laboratory for Vaccine-Preventable Diseases, National Institute of Public Health and the Environment, Bilthoven, The Netherlands; e Department for Primary and Community Care, Radboud University Medical Center, Nijmegen, The Netherlands; f Department of Viroscience, Erasmus University Medical Center, Rotterdam, The Netherlands; g Foundation Stichting Down Syndroom, Meppel, The Netherlands; h Sanquin Research and Landsteiner Laboratory, Amsterdam University Medical Center, University of Amsterdam, Amsterdam, The Netherlands; i Department of Biomolecular Mass Spectrometry and Proteomics, Utrecht Institute for Pharmaceutical Sciences and Bijvoet Center for Biomolecular Research, Utrecht University, Utrecht, The Netherlands; j Vaccine Research Center, National Institute of Allergy and Infectious Disease, Bethesda, MD, USA; k Tranzo, Tilburg School of Social and Behavioral Sciences, Tilburg University, Tilburg, The Netherlands; l Jeroen Bosch Academy Research, Jeroen Bosch Hospital, ‘s-Hertogenbosch, The Netherlands; m Department of Pediatrics, Alrijne Hospital, Leiderdorp, The Netherlands; n Department of Epidemiology of Microbial Diseases, Yale School of Public Health, New Haven, CT, USA

**Keywords:** Down syndrome, COVID-19, SARS-CoV-2, mRNA and vector vaccines, T cells, IFNγ

## Abstract

Down syndrome (DS) is associated with immune dysfunction, which led to higher hospitalization and mortality rates during the COVID-19 pandemic. We previously showed that antibody concentrations were lower in adults with DS after primary SARS-CoV-2 vaccination. However, knowledge on cellular vaccine-induced responses in DS is limited. Here, we investigated the T-cell response induced by SARS-CoV-2 vaccination in adults with DS. We included adults with DS and healthy controls (HC) between 18 and 64 years following primary (mRNA and vector) and booster (mRNA) SARS-CoV-2 vaccination. Using flow cytometry, SARS-CoV-2-specific T cells were analyzed after spike peptide re-stimulation. Additionally, interferon-gamma (IFNγ) production by SARS-CoV-2-specific T cells was measured with an IFNγ release assay (IGRA) after antigen stimulation. We observed major deficits in naive CD4^+^ and CD8^+^ T cells in adults with DS as previously described. However, overall there was no difference in the percentage of SARS-CoV-2-specific T cells or IFNγ production by these cells after primary vaccination, albeit with vaccine-specific differences. IFNγ concentrations measured by IGRA after primary vaccination were comparable between DS and HC. Booster vaccination increased SARS-CoV-2-specific T cells in HC but not in DS, while IFNγ release (IGRA) increased to similar concentrations. In conclusion, we show that while functional T-cell responses (IFNγ production) are comparable after primary and booster vaccination, the magnitude (percentage of SARS-CoV-2-specific T cells) may be lower after mRNA vaccination. We previously showed lower antibody concentrations and profound abnormalities in the naive T-cell compartment, warranting further investigation into T-cell-B-cell-interactions after vaccination in adults with DS.

**Clinical trial registration**: NCT05145348 https://www.clinicaltrials.gov/study/NCT05145348?locStr=Netherlands&country=Netherlands&cond=Down%20Syndrome&term=SARS-CoV-2%20Vaccination&rank=1.

**Start date**: 2021–02-03.

## Background

Down syndrome (DS) is the most common chromosomal aberration worldwide, with an incidence of 9.9 per 10,000 livebirths in the Netherlands in 2018.^1^ DS is associated with comorbidities, including but not limited to variable intellectual disability, congenital heart disease, thyroid disease, autoimmunity and immune dysregulation.^2,3^ This immune dysregulation affects both humoral and cellular compartments of the immune system, resulting in lower antibody production, reduced counts of switched B cells, and mild to moderate T-cell lymphopenia, with less naive T cells.^4,5^ The lower naive T-cell counts in individuals with DS are thought to result from reduced thymic output, leading to a shift toward higher memory T-cell frequencies.^6^ Furthermore, individuals with DS are more prone to infections, especially respiratory infections, that are also associated with severe outcomes.^7^ This was also evident during the coronavirus disease 2019 (COVID-19) pandemic. Individuals with DS displayed a fourfold increase in hospitalization rates and up to 10 times-higher mortality rates after severe acute respiratory syndrome coronavirus 2 (SARS-CoV-2) infection.^8,9^ Even after one or two SARS-CoV-2 vaccinations, people with DS were still 12.7 times more likely to develop severe outcomes after SARS-CoV-2 infection.^10^ This clearly highlights the importance of investigating vaccine-induced immune responses in DS.

Vaccine responses in individuals with DS have only sporadically been studied and most studies focused on small cohorts of children, looking solely at antibody production. The results varied significantly depending on vaccine type, pathogen, and sometimes even serotype. However, a key finding is that individuals with DS respond suboptimal to certain vaccines. Children with DS showed lower antibody titers after influenza A^11^ or hepatitis B^12^ vaccination compared to children without DS. For adults, it has been shown that they develop lower antibody titers after hepatitis B vaccination, especially with increasing age,^13^ and have a diminished response to influenza A vaccination measured by hemagglutinin inhibition assay.^14^ For SARS-CoV-2 vaccines, most studies also mainly focused on the humoral immune response and found lower anti-spike or anti-receptor binding domain antibodies titers after two vaccinations in DS compared to healthy controls (HC),^15–17^ suggesting a less robust immune response in people with DS. However, knowledge about the cellular vaccine-induced immune response in DS is still limited. As individuals with DS have less naive T cells, it remains elusive whether vaccinations induce similar vaccine-induced T cells in percentage and function as observed in HC. Here, our objective was to characterize the T-cell response in adults with DS and HC following two primary SARS-CoV-2 vaccinations (mRNA or vector) and a third (mRNA) booster dose to better understand cellular vaccine-induced immunity in individuals with DS.

## Methods

### Study design and participants

**Figure 1.**
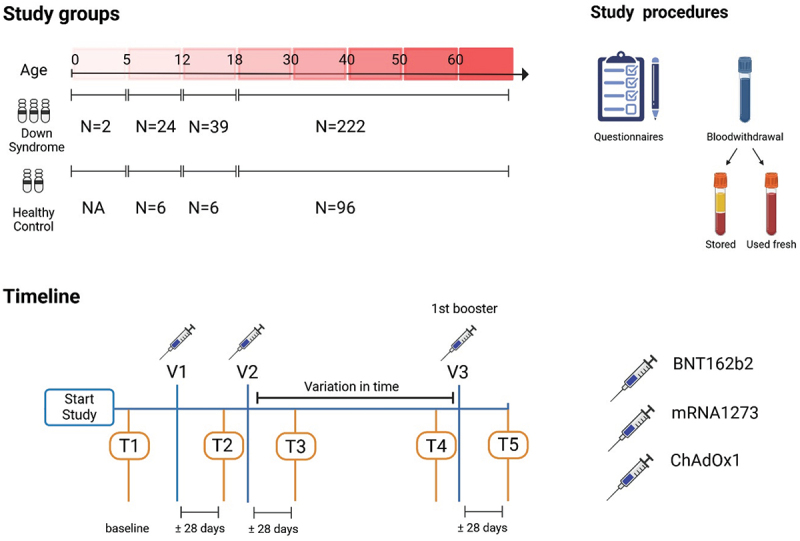
PRIDE study design. Pride is an observational, prospective cohort study performed in the Netherlands to investigate the SARS-CoV-2 vaccine-induced immune response in adults and children with Down Syndrome (DS) compared to healthy controls (HC).^15^ All adult participants received two vaccinations of BNT162b2 (Pfizer-BioNTech, 3–6 weeks interval), mRNA-1273 (Moderna, 4–6 weeks interval) or ChAdOx1 (AstraZeneca, 10–14 weeks interval) as part of the Dutch national immunization program. Approximately 5–9 months after primary vaccination, participants received, on a voluntary basis, an mRNA booster vaccination, either BNT162b2 or mRNA-1273. Blood samples were collected at five timepoints: T1, before the first vaccination (baseline), T2 and T3, approximately 28 days after the first and second vaccination, respectively. T4 was collected within three weeks before the booster vaccination and T5 approximately 28 days after the booster vaccination. Blood was used directly to determine absolute leukocyte counts and to perform the interferon gamma release assay (IGRA). Serum was stored at −80°C and peripheral mononuclear cells (PBMCs) in liquid nitrogen for later analysis.

This project is part of the Prospective Monitoring of Antibody Response Following COVID-19 Vaccination in Patients with Down Syndrome (PRIDE) study.^15^ In short, inclusion criteria were a clinical diagnosis of DS for DS participants and not for healthy controls (HC) in this observational, prospective study. Exclusion criteria consisted of active malignancy, treatment for malignancy in the past 3 months, receipt of an organ transplant, and human immunodeficiency virus infection. HC participants with any condition requiring regular visits to a healthcare provider were also excluded. Clinical variables were collected via questionnaires. An overview of the study design and time points can be found in Figure 1. We only included adults for T-cell analysis. The University Medical Centre Utrecht medical research ethics committee (NL76336.041.21) approved the PRIDE study and all participants and/or legal representatives provided written informed consent before inclusion. Participants were excluded from further analyses in case of a natural SARS-CoV-2 infection based on a positive anti-S IgG concentration at T1 (>10.08 BAU/ml) or a positive anti-N IgG concentration at any time point (>14.3 BAU/ml).^18^

Created in BioRender. Hensen, L. (2025) https://BioRender.com/gn20rmz..

### Reagents

All reagents used in this study are listed in Supplementary Table S1.

### Absolute leukocyte counts

Absolute leukocyte counts and differentiation were determined using Abbott Alinity hq analyzer.

### Flow cytometry

#### Sample selection

We measured N = 29 DS and N = 28 HC samples for phenotyping and the activation induced marker (AIM) assay at T3. A flowchart illustrating the sample selection for T3 can be found in Supplementary Figure S1. For analysis of the booster vaccination samples, participants were selected based on peripheral blood mononuclear cells (PBMC) availability at T4 (DS N = 22, HC N = 13) and T5 (DS N = 23, HC N = 15) and excluded in case of a previous natural SARS-CoV-2 infection at T1, T2, T3, T4 or T5 as described in the study design. Due to limited sample availability, no further selection criteria were taken into account.

#### T-cell and B-cell phenotyping

PBMCs were thawed and Fc receptors blocked with normal mouse serum for 10 minutes at 4°C. Cells were stained with a viability dye for 20 minutes at 4°C. Subsequently, PBMCs were surface stained for CD3, CD4, CD8, CCR7, CD45RO, and CD31 for T cells and CD19 for B cells. All samples were acquired on a BD LSR Fortessa and data analyzed in FlowJo. The detailed flow cytometry protocol can be found in Supplementary Figure S2 and gating details are provided in Supplementary Figure S3.

#### AIM assay

1 × 10^6 PBMCs were stimulated with SARS-CoV-2 spike peptide pool (1 µg/mL per peptide), an equimolar amount of dimethyl sulfoxide (DMSO) or dynabeads for 20–24 hours in 200 µL. The peptide pool consisted of 315 15-mer peptides, overlapping by 11 amino acids across the full length of the wild-type SARS-CoV-2 spike protein, ensuring HLA-independent stimulation.^19^ The peptide pool is suited for CD4^+^ T-cell reactivation, and to a limited extend CD8^+^ T cells. The final 3 hours of incubation, Golgistop and CD137 antibody were added in culture. PBMCs were stained for flow cytometry analysis as described for T-cell phenotyping. Surface staining was performed for CD3, CD4, CD8, CD45RO, CD134, and CD69. Additionally, cells were fixed and permeabilized for 30 minutes at 4°C and intracellular stained for IFNγ for 20 minutes at 4°C. Samples were acquired on a BD LSR Fortessa and analyzed in Flowjo. The complete protocol is depicted in Supplementary Figure S4. SARS-CoV-2-specific memory T cells (AIM^+^ T cells) were defined as CD134^+^CD137^+^ for CD4^+^ T cells and CD69^+^CD137^+^ for CD8^+^ T cells. The DMSO-stimulated sample was used to set the cutoff gate for activation markers. AIM^+^ T cells are either depicted as background subtracted or stimulation index (SI), calculated by dividing specific activation (S-peptide stimulation) over background activation (DMSO). Negative AIM^+^ percentages after background subtraction were set to 0. An SI of 2 or higher was considered a positive vaccine-induced T-cell response.^20^

### Interferon-gamma release assay (IGRA)

#### Sample selection

We measured IFNγ production in N = 49 DS and N = 23 HC samples. An overview of the sample selection for T3 can be found in Supplementary Figure S5. Samples from T4 and T5 were selected similarly. For T4, we measured N = 39 DS and N = 11 HC samples, and for T5 N = 39 DS and N = 12 HC.

#### IFNγ detection

Fresh whole blood was re-stimulated with SARS-CoV-2 antigen using the Quan-T-Cell SARS-CoV-2 kit (EUROIMMUN) according to the manufacturer’s protocol. Isolated plasma was harvested and stored at −80°C for subsequent detection of IFNγ by enzyme-linked immunosorbent assay (ELISA) with the commercial IFNγ ELISA kit Quan-T-Cell ELISA (EUROIMMUN) according to the manufacturer’s instructions. The optical density 450 (OD450) was measured on the Clariostar and corrected by subtracting background signal measured in the OD650 channel. We constructed a 4-parameter dose response curve based on the standard ODs in Prism 10 and extrapolated the unknown concentrations in mIU/mL. An IFNγ concentration between 100–200 mIU/mL was considered a borderline vaccine reaction, and ≥200 mIU/mL a positive reaction.

### Statistical analysis

Statistical analysis were performed in GraphPad Prism 10. Patient characteristics were described as median, interquartile range (IQR) and percentages as appropriate. Datasets with >30 samples were tested for normality. Datasets < 30 samples were assumed non-Gaussian distributed. Mann-Whitney U tests were performed for non-Gaussian, unpaired data and Wilcoxon test for paired data. Kruskal-Wallis test was used to determine significance in case of > two groups. Spearman’s Rank-Order Correlation was calculated to determine correlation for not Gaussian distributed data. Categorical variables were compared between groups using the Chi-square test or Fisher’s exact test. The Chi-square test was applied when all expected cell counts were ≥5; otherwise, Fisher’s exact test was used. Data was considered significant if *p* < .05.

## Results

### Baseline characteristics of participants

The selected DS and HC participants for analyses after primary vaccination (T3) were similar in age and sex. Further characteristics are presented in Table 1. This selected cohort is a representation of the PRIDE cohort, confirmed by similar observed trends, namely lower lymphocyte counts and lower anti-S1 antibody titers in DS samples (Supplementary Figure S6(A,B)).

**Table 1.** Baseline characteristics T3 flow cytometry cohort.

	DS (N = 29)	HC (N = 28)	*p*^a^
Age (median, IQR^b^)	29 (24–51)	30 (26–56)	.4436
Male sex (n, %)	15 (52%)	12 (43%)	.5027
Ethnicity North West European (n, %)	27 (93%)	28 (100%)	.4912
Smoking (n, %)	0 (0%)	1 (4%)	.4912
SARS-CoV-2 vaccine type primary series BNT162b2 (n, %) mRNA-1273 (n, %) ChAdOx1 (n, %)	16 (55%) 3 (10%) 10 (35%)	13 (46%) 4 (14%) 11 (39%)	.8089
Medical history Thyroid disease (n, %) Celiac disease (n, %) Diabetes mellitus^c^ (n, %)	8 (28%) 1 (4%) 2 (7%)	0 (0%) 0 (0%) 0 (0%)	>.9999

^a^Significance tested with Mann-Whitney U, Chi-square or Fisher’s exact test as described in the methods.

^b^IQR, interquartile range.

^c^Including DM type 1 and type 2.

### Characterization of T-cell and B-cell compartments

**Figure 2.**
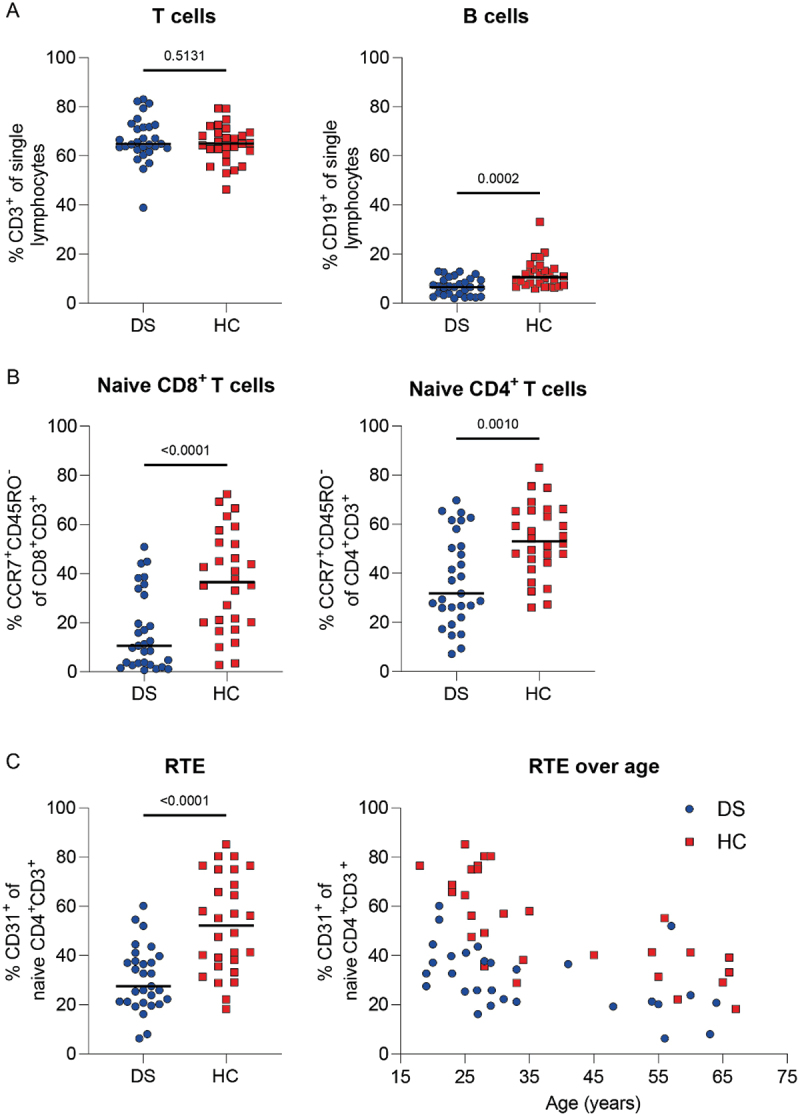
Phenotypic differences in the T-cell and B-cell compartments of adults with DS at T3. (A) Percentage T and B cells. (B) Naive CD8^+^ and CD4^+^ T cells. (C) Percentage recent thymic emigrants (RTEs) and correlation of RTEs with age in DS and HC. Significance in (A–C) was determined using Mann-Whitney test and the median is shown as a black line. The correlation with age in (C) was determined by Spearman’s rank correlation coefficient. DS N = 29 and HC N = 28 for (A–C).

We confirmed the generally known T-cell and B-cell abnormalities in DS. Individuals with DS had lower absolute lymphocyte counts at T3 compared to HC (DS = 1.4210^9/L vs HC = 1.9310^9/L, *p* < .0001, Supplementary Figure S7). No difference in the percentage of CD3^+^ T cells was observed, however significant lower percentages of CD19^+^ B cells were present in DS (Figure 2(A)). Percentages of naive CD8^+^ and CD4^+^ T cells were significantly lower in DS (Figure 2(B)), while percentages of memory subpopulations were significantly higher (data not shown). Reduced naive CD4^+^ T-cell percentages in DS were further characterized by significant lower recent thymic emigrants percentages (RTE, CD31^+^ of naive CD4^+^ T cells, Figure 2(C)). In both groups, a negative correlation between RTEs and age was detected (DS Spearman *r* = −0.65, *p* = .0018, HC Spearman *r* = −0.79, *p* < .0001). Overall, we observed distinct differences in the T-cell and B-cell adaptive immune compartments of individuals with DS.

### SARS-CoV-2 vaccine-induced T-cell response after primary vaccination

**Figure 3.**
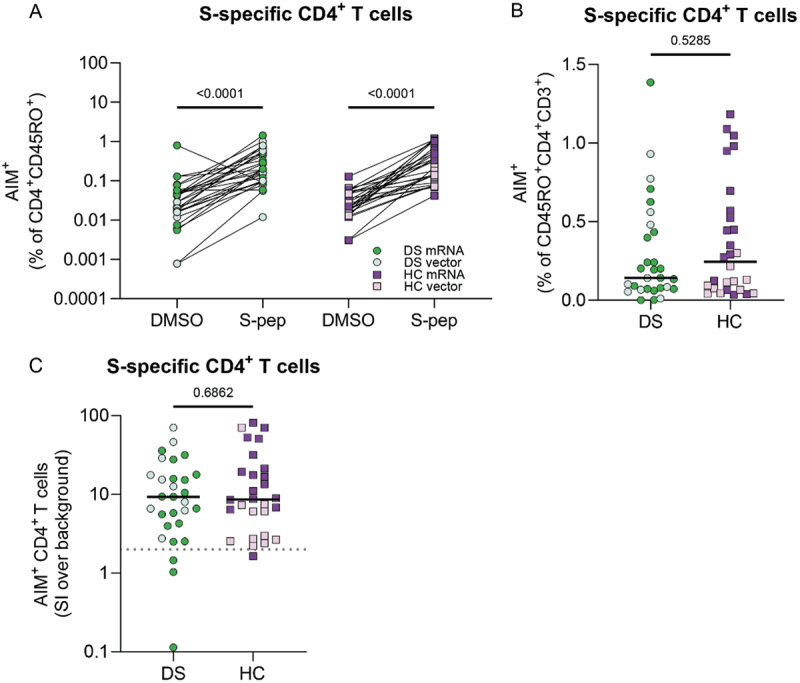
Similar SARS-CoV-2-specific CD4^+^ T cells in DS after two vaccinations. (A) percentage of AIM^+^ cells within the CD4^+^CD45RO^+^ subset after DMSO or spike peptide stimulation. AIM^+^CD4^+^ cells are defined as CD134^+^CD137^+^. (B) Percentage of AIM^+^ cells within the CD4^+^CD45RO^+^ subset after subtraction of the DMSO background. (C) Stimulation index (SI) of AIM^+^CD4^+^ T cells, calculated by dividing specific activation over background activation. An SI of 2 or higher is considered a positive T-cell response (dashed line). Significance in (A) was determined using Kruskal-Wallis test. Significance in (B and C) was determined using Mann-Whitney tests and the median is shown as a black line. DS N = 29 and HC N = 28 for (A–C).

To investigate the presence of SARS-CoV-2-specific T cells after primary SARS-CoV-2 vaccination, we stimulated PBMCs with ancestral spike peptides (S-pep) to identify these T cells. There was no difference in activation of CD4^+^ T cells or CD8^+^ T cells between DS and HC after a-specific stimulation (Supplementary Figure S8(A,B)), indicating that the intrinsic activation capacity of T cells was not hampered in DS. For most samples, both DS and HC, we observed a higher percentage AIM^+^CD4^+^ T cells after S-pep stimulation compared to background (DMSO) (Figure 3(A)). S-pep stimulation did not lead to a significant increase of AIM^+^CD8^+^ T cells compared to background in DS, while for HC this was the case (Supplementary Figure S8(C)). After background subtraction, there was no significant difference between DS and HC in the percentage of AIM^+^CD4^+^ T cells (Figure 3(B)) or AIM^+^CD8^+^ T cells (Supplementary Figure S8(D)). Since the percentage of AIM^+^CD8^+^ T cells after background subtraction was low, we only continued with the analysis of SARS-CoV-2-specific CD4^+^ T cells. To measure the induction of AIM^+^CD4^+^ T cells by primary vaccination, we calculated the stimulation index (SI). There was no difference in the SI of AIM^+^CD4^+^ T cells between DS and HC at T3 (Figure 3(C)).

**Figure 4.**
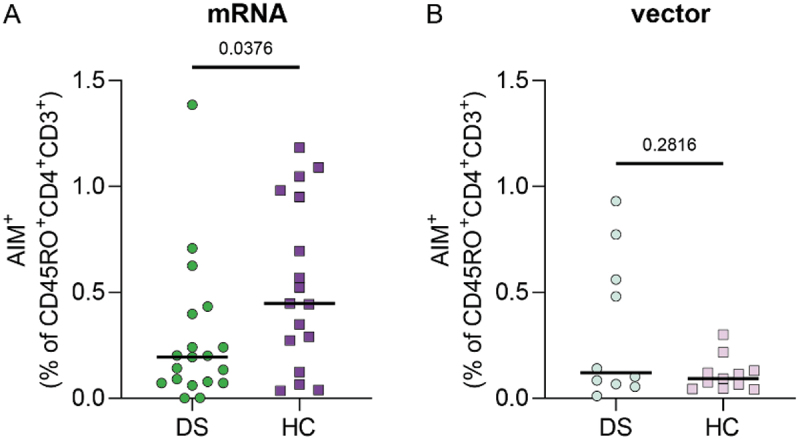
Comparison of mRNA and vector vaccination in DS and HC based on percentage of S-specific CD4^+^ T cells. (A) Difference between DS and HC after mRNA (DS N = 19, HC N = 17) and (B) vector (DS N = 10, HC N = 11). Shown as the percentage of AIM^+^ cells within the CD4^+^CD45RO^+^ subset after S-peptide stimulation with background (DMSO) subtraction. Significance in (A–B) was determined using Mann-Whitney tests and the median is shown as a black line.

Interestingly, when comparing mRNA and vector vaccinated groups separately, we found lower percentages of AIM^+^CD4^+^ T cells after mRNA vaccination in DS compared to HC (DS = 0.19%, N = 19 vs HC = 0.45%, N = 17, *p* = .034, Figure 4(A)). There was no difference in AIM^+^CD4^+^ T-cell percentages between the participants who received vector vaccination (Figure 4(B)). Together, these results show that overall, primary SARS-CoV-2 vaccination induces similar T-cell responses in DS and HC. However, differences are observed when zooming in per vaccine type.

### IFNγ production by SARS-CoV-2-specific T cells

**Figure 5.**
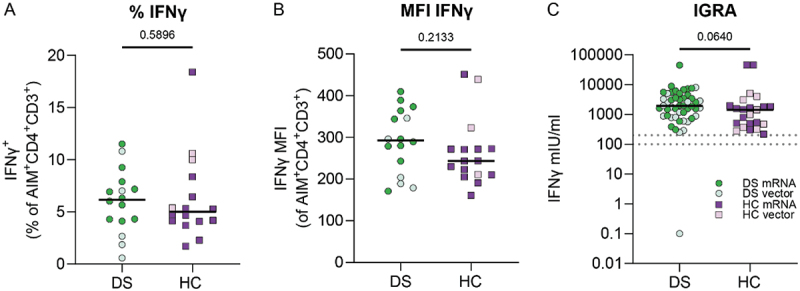
Comparable IFNγ production by SARS-CoV-2-specific T cells in DS and HC after two vaccinations. (A) Percentage of IFNγ^+^ AIM^+^CD4^+^ T cells and (B) mean fluorescence intensity (MFI) of IFNγ^+^ AIM^+^CD4^+^ T cells after two SARS-CoV-2 vaccinations (DS N = 16, HC N = 16). (C) IFNγ (mIU/ml) production by SARS-CoV-2-specific T cells measured by IGRA (DS N = 49, HC N = 23). An IFNγ concentration between 100–200 mIU/mL was considered a borderline vaccine reaction, and >200 mIU/mL a positive reaction, indicated by the dashed lines. Significance in (A–C) was determined using Mann-Whitney tests and the median is shown as a black line.

Next, we evaluated the function of vaccine-induced SARS-CoV-2-specific CD4^+^ T cells in DS by measuring IFNγ production after S-pep stimulation. There were no differences in the percentage of IFNγ^+^AIM^+^CD4^+^ T cells (Figure 5(A)) as well as in the IFNγ production (mean fluorescence intensity, MFI, Figure 5(B)) between DS and HC.

In addition, we measured IFNγ production by all vaccine-induced specific T cells using IGRA. SARS-CoV-2-specific T cells of adults with DS produced comparable IFNγ levels to HC at T3 (Figure 5(C)). When comparing the IFNγ production per vaccine type, these results held true (Supplementary Figure S9(A,B)). In conclusion, SARS-CoV-2-specific T cells of individuals with DS and HC produce similar quantities of IFNγ.

### T-cell response after SARS-CoV-2 booster vaccination

**Figure 6.**
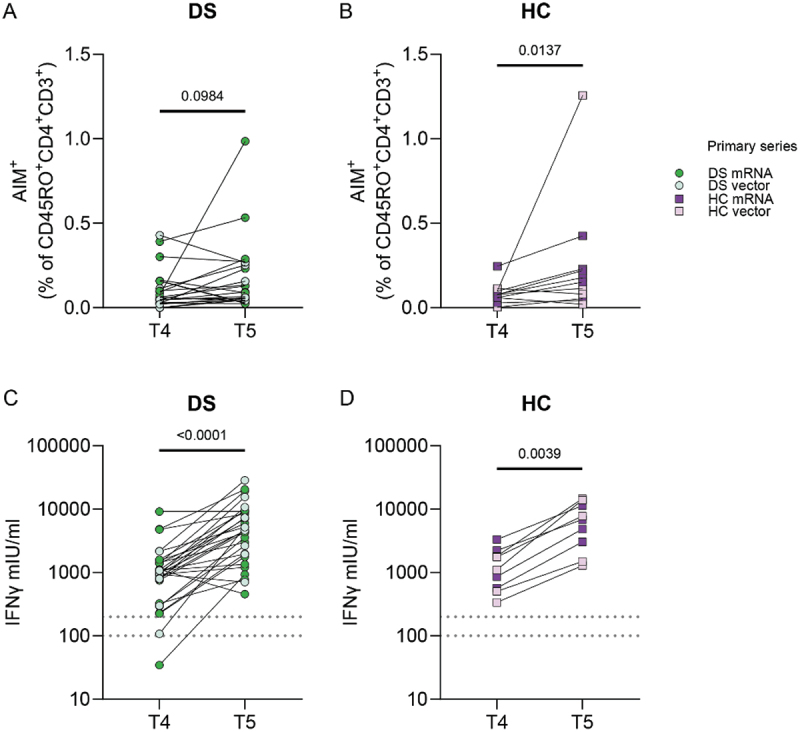
Comparable effect of SARS-CoV-2 booster vaccination in DS and HC. (A) Percentage of AIM^+^ cells within the CD4^+^CD45RO^+^ subset after subtraction of the DMSO background in paired samples before (T4) and after (T5) booster vaccination for DS (N = 21) and (B) HC (N = 11). AIM^+^CD4^+^ cells are defined as CD134^+^CD137^+^. Participants were grouped in the mRNA or vector group based on their primary SARS-CoV-2 vaccine regimen. (C) IFNγ (mIU/ml) production by SARS-CoV-2 specific T cells at T4 and T5 for DS (N = 30) and (D) HC (N = 9). An IFNγ concentration between 100–200 mIU/mL was considered a borderline vaccine reaction, and >200 mIU/mL a positive reaction, indicated by the dashed lines. Significance in (A–D) was determined using Wilcoxon tests.

During the pandemic, an mRNA booster vaccination was implemented for all individuals in the Netherlands, regardless of prior vaccine type. To investigate the cellular response after booster vaccination in DS, we evaluated SARS-CoV-2-specific T cells before (T4) and after (T5) booster vaccination. Baseline characteristics of included DS and HC participants for these assays are presented in Supplementary Table S2 and S3. In DS, the percentage of AIM^+^ CD4^+^ T cells did not increase after booster vaccination (T4 vs T5, Figure 6(A)), whereas HC showed a significant booster-induced increase (Figure 6(B)). When analyzed per vaccine type, this increase was mainly seen in primary mRNA vaccinated individuals (*p* = .0312), not primary vector vaccinated HC (*p* = .4375). However, when comparing DS and HC per timepoint (T4 and T5), no significant differences were observed between both groups (Supplementary Table S10(A,B)).

Neither the percentage of IFNγ^+^AIM^+^CD4^+^ T cells, nor the IFNγ production by AIM^+^CD4^+^ T cells (MFI) increased after booster vaccination in DS and HC (data not shown). However, IGRA analyses showed a clear increase of IFNγ production from T4 to T5 in both DS and HC samples (Figure 6(C,D)). There was no difference in the IFNγ concentration between DS and HC at both timepoints (Supplementary Figure S11(A,B)). These results held true when analyzing IFNγ concentrations for mRNA and vector vaccinated individuals separately (data not shown). Thus, while booster vaccination does not increase the percentage of SARS-CoV-2-specific T cells in DS, it does induce a similar, significant increase in IFNγ production as observed in HC.

## Discussion

Down syndrome is associated with immune dysregulation and in line with previous studies, we observed lower naive CD4^+^ and CD8^+^ T cells, B-cell lymphopenia and lower CD4^+^ RTE within DS participants of the PRIDE study.^4–6,21^ Nonetheless, these differences do not seem to impact the vaccine specific response on a cellular level, as we observed similar percentages of SARS-CoV-2-specific CD4^+^ T cells in DS and HC after primary vaccination (mRNA and vector combined) and after an mRNA-based booster vaccination. Furthermore, we found an overall similar functionality of SARS-CoV-2-specific T cells based on IFNγ production. Booster vaccination did not lead to an increase of SARS-CoV-2-specific CD4^+^ T cells in DS, however it increased IFNγ production by T cells in both DS and HC to equal concentrations.

We are the first to investigate the T-cell response after both mRNA and vector-based vaccines in individuals with DS. A previous study observed that individuals with DS mount an effective T-cell response after two or three (booster) mRNA vaccinations, based on the percentage of T-cell responders versus non-responders.^22,23^ However, the magnitude of the T-cell response was not investigated. Re-analysis of the raw data of this study revealed significant lower percentages of SARS-CoV-2-specific T cells after two mRNA vaccinations in the DS samples compared to HC (Supplementary Figure 12). These findings are in line with our data on mRNA vaccination, underlining a lower T-cell response in DS after two mRNA vaccinations. For the vector-based vaccine ChAdOx1, we observed no difference between DS and HC after two vaccinations. Since, Esparcia-Pinedo *et al*. only included mRNA-vaccinated individuals (except for one participant), validation of our results in other cohorts might help elucidate vaccine-type specific differences in DS. Despite the lower percentages after mRNA vaccination, SARS-CoV-2-specific T cells from adults with DS were functionally similar to T cells from HC (based on IFNγ production), suggesting the induction of an adequate vaccine response by mRNA vaccination. Moreover, since the percentages of SARS-CoV-2-specific CD4^+^ T cells and the functional response are similar in DS and HC when grouping both mRNA and vector vaccinated individuals together, we conclude that overall individuals with DS respond normally to SARS-CoV-2 vaccination on a T-cell level. Measurements of other key cytokines involved in T effector functions during viral infections, such as tumor necrosis factor alpha and granzyme B,^24^ might further validate the efficacy of the T-cell response observed in DS after primary SARS-CoV-2 vaccination.

We were able to detect SARS-CoV-2-specific CD4^+^ T cells, while detection of specific CD8^+^ T cells was limited, likely due to the length of the used peptides.^25^ The peptide 15-mers are ideal in length to restimulate CD4^+^ T-cell memory responses, while for CD8^+^ T-cell activation, the peptides first need to be processed by antigen presenting cells (APCs) present in the PBMCs, which might vary considerably per participant. Consequently, the effectiveness of the CD8^+^ T-cell response following vaccination was less well investigated here and should be further explored since they are critical for eliminating virus-infected cells and control the infection,^24,26^ thereby preventing severe disease. Future studies could implement shorter peptide lengths, that don’t need processing by APCs, to optimally detect CD8^+^ T-cell responses. We now focused on CD4^+^ T cells, given their central role in activating B cells, thereby driving antibody production,^27^ which underpins the preventive action of vaccines. The function of these SARS-CoV-2-specific CD4^+^ T cells in DS was similar to HC as shown by IFNγ production measured by flow cytometry after primary vaccination. Using IGRA, we measured the total CD4^+^ and CD8^+^ T-cell response, which was as effective in DS compared to HC, after primary vaccination. This suggests that individuals with DS are also capable of mounting an effective CD8^+^ T-cell response following SARS-CoV-2 vaccination. Moreover, we observed a significant increase of IFNγ levels after booster vaccination with IGRA, but did not observe a booster effect on IFNγ production by SARS-CoV-2 CD4^+^ T cells using flow cytometry, which might indicate that booster vaccination mainly boosts CD8^+^ and not CD4^+^ T cells. This likely reflects a qualitative improvement in T-cell functionality rather than expansion of the antigen-specific pool. However, the contribution from CD8^+^ T cells remains speculative in the absence of detailed cytokine profiling.

Individuals with DS have lower antibody titers after primary SARS-CoV-2 vaccination.^15–17^ Specifically for the antibody response after mRNA vaccination, it was also shown that age had a negative effect in DS.^15^ We did not observe an effect of age on the percentage of AIM^+^ CD4^+^ T cells (data not shown). This illustrates that different factors play a role in mounting an effective humoral and cellular immune response in DS. T follicular helper (Tfh) cells lay at the crossroads of the cellular and humoral immune response and may play a role in the suboptimal induced vaccine responses in DS. They play an essential role in B-cell development and long-term humoral immunity.^28^ While Esparcia-Pinedo and colleagues found no difference in circulating spike-specific Tfhs after primary vaccination,^22^ Tfhs have been shown to be reduced in both tonsils and in the circulation of children with DS,^29^ with a skewed CXCR3^+^ Tfh1 phenotype suggestive of poor B cell helper function.^28^ Additionally, the lower percentage of AIM^+^ CD4^+^ T cells in adults with DS after mRNA vaccination might result in less interactions with B cells, contributing to lower antibody titers. Future research on the interplay between the humoral and cellular immune responses, like exploring the function of Tfhs, will help us better understand vaccine-induced immune responses in DS.

A key strength of our study is the inclusion of individuals vaccinated with mRNA and vector-based vaccines, as well as the comprehensive assessment of vaccine-induced T-cell responses using different assays, including both descriptive and functional approaches. However, a limitation is the relatively small sample size, especially for vector vaccinated individuals and booster analysis. We have calculated the statistical power post hoc for four comparisons (Figure 4(A,B); comparison of mRNA and vector vaccination in DS versus HC based on SARS-CoV-2 specific CD4^+^ T cells, and Figure 6(A,B); comparison of SARS-CoV-2 specific CD4^+^ T-cell percentages before and after booster vaccination within DS and HC participants) because these comparisons had the smallest group sizes. The statistical power ranges between 30–53%. We acknowledge that these smaller subgroup sizes reduce the statistical power of the comparisons and therefore the chances to detect meaningful differences, particularly for the booster analysis. To best study the effect of DS on the vaccine-induced immune response, for our T3 flow cytometry analysis, we excluded major comorbidities such as congenital heart disease requiring surgery. Heart surgery might partially or completely remove the thymus, and thereby negatively impact different T-cell compartments.^30^ However, up to 50% of individuals with DS suffer from congenital heart disease of varying severity,^31^ thus not all needing cardiac surgery, but this may limit the potential for generalization of our dataset. However, the DS and HC samples analyzed at T3 showed representative trends (absolute lymphocyte counts, T-cell phenotypes and anti-S1 antibody titers) as observed by others,^5,15^ suggesting that our cohort remains valid for addressing the study objective. Nonetheless, future studies should aim to include and stratify participants based on common comorbidities such as congenital heart disease to better reflect the broader DS population. Furthermore, while DS and HC samples were age and sex matched, the HC group lacked comorbidities and may not be fully comparable in terms of biological age, given the premature aging and immunosenescence observed in DS.^32–35^ Due to sample availability, we were not able to follow the same participants after primary vaccination and booster vaccination, limiting both intra-individual comparisons and determination of the decay of the response in DS an HC. A limitation of the PRIDE study is limited data on the clinical effect of vaccination on disease severity and hospitalization. Despite the high number of DS participants included, we observed low numbers of breakthrough infections resulting in hospitalization. Therefore, we were not able to correlate the cellular vaccine-induced immune response to clinical effectiveness. However, other clinical studies provide important context, showing that COVID-19 vaccination markedly reduced mortality in DS (odds ratio: 0.0002)^36^ and was associated with very few breakthrough infections (1% in a cohort of 1,973 vaccinated individuals).^37^ These findings suggest that, despite reduced antibody responses, the comparable functional T-cell responses we observed between DS and HC are likely to contribute to the protective effect of vaccination in this population.

In conclusion, while adults with DS exhibit clear abnormalities in their T-cell compartment, we observed that the functional T-cell response (IFNγ production) is comparable after primary vaccination, while the percentage of SARS-CoV-2-specific CD4^+^ T cells may be lower after mRNA vaccination. This suggests that, with regard to future pandemics, mRNA and vector-based vaccines would induce functionally effective T-cell responses in DS. Nonetheless, the lower antibody titers in adults with DS after primary vaccination – which might be linked to the lower percentage of SARS-CoV-2 specific CD4^+^ T cells after mRNA vaccination – as observed previously^15–17^ should not be overlooked, nor that DS remains a risk factor for severe disease even after vaccination.^10^ Booster vaccination increased T-cell function in DS to levels similar to those observed in HC, indicating that implementing booster vaccinations in vaccine schedules for individuals with DS may be an effective strategy to enhance protection in this vulnerable population. Future research focused on vaccine-induced CD8^+^ T-cell responses, Tfhs and the interaction between the cellular and humoral immune response in DS could further tailor and improve vaccine strategies in DS.

## Supplementary Material

251015_Hensen_SupplementaryFigures_revised_clean.docx

## Data Availability

The data generated and/or analyzed during this study are available upon request from the corresponding author.
