# Supplementary material for: T-cell responses to SARS-CoV-2 vaccinations in adults with Down syndrome – a prospective cohort study
Source: Hum Vaccin Immunother. 2025 Nov 6;21(1):2583416. doi: 10.1080/21645515.2025.2583416 (PMC12599571; doi:10.1080/21645515.2025.2583416)
Supplement: 251015_Hensen_SupplementaryFigures_revised_clean.docx [file KHVI_A_2583416_SM1148.docx]

**Supplemental information**

**Supplemental Table 1: overview of materials.**

| **Antibodies** | | | | | | | |
| --- | --- | --- | --- | --- | --- | --- | --- |
| *Target* | *Label* | *Vendor* | *Clone* | *Catalog#* | *Usage* | *RRID* |  |
| Fixable Viability Dye | eFluor506 | eBioscience | N.A. | 65-0866-14 | 1000x | N.A. |  |
| CD3 | AF700 | Biolegend | UCHT1 | 300424 | 50x | AB_493741 |  |
| CD4 | APC-eF780 | eBioscience | RPA-T4 | 47-0049-42 | 200x | AB_1272044 |  |
| CD8 | BV605 | Biolegend | SK1 | 344742 | 50x | AB_2566513 |  |
| CCR7 | APC | Biolegend | G043H7 | 353214 | 40x | AB_10917387 |  |
| CD45RO | ECD | Beckman Coulter | UCHL1 | B49192 | 25x | AB_3073671 |  |
| CD31 | FITC | BD Biosciences | WM59 | 555445 | 50x | AB_395838 |  |
| CD19 | APC-eF780 | eBioscience | HIB19 | 47-0199-42 | 20x | AB_1582230 |  |
| CD137 | PE | BD Biosciences | 4B4-1 | 555956 | 50x | AB_396252 |  |
| CD134 | PerCP-Cy5.5 | Biolegend | Ber-ACT35 | 350010 | 25x | AB_10901161 |  |
| CD69 | APC | Biolegend | FN50 | 310910 | 100x | AB_314845 |  |
| IFN-γ | PE-Cy7 | BD Biosciences | 4S.B3 | 557844 | 200x | AB_396894 |  |
| **Reagents** | | | | |  | | |
| *Name* | | *Vendor* | | *Catalog#* | *Usage* | |  |
| RPMI 1640 medium | | Gibco | | 12017599 | N.A. | |  |
| Normal mouse serum | | Bioconnect | | 88-NM35 | 25x | |  |
| FcR blocking reagent human | | Miltenyi Biotec | | 130-059-901 | 2% | |  |
| FoxP3 transcription factor staining buffer set | | eBioscience | | 00-5523-00 | According to manufacturer | |  |
| PepMix SARS-CoV-2 (Spike Glycoprotein) | | JPT | | PM-WCPV-S-1 | 1 µg/mL per peptide | |  |
| DMSO | | Sigma-Aldrich | | D5879 | Equimolar as peptide pool | |  |
| Dynabeads Human T-activator CD3/CD8 | | Invitrogen | | 10587973 | 1 bead per 5 PBMCs | |  |
| GolgiStop | | BD Bioscience | | 554724 | 1500x | |  |
| Brilliant Stain buffer | | BD Bioscience | | 563794 | 8% | |  |
| Quan-T-Cell SARS-CoV-2 | | EUROIMMUN | | ET 2606-3003 | According to manufacturer | |  |
| Quan-T-Cell ELISA | | EUROIMMUN | | EQ 6841-9601 | According to manufacturer | |  |
| **Devices and software** | | | | | | | |
| *Name* | | *Source* | | | | |  |
| Clariostar plate reader | | BMG LABTECH | | | | |  |
| BD LSR Fortessa (4-laser) flow cytometer | | BD Bioscience | | | | |  |
| FlowJo V10.8.1 | | BD Bioscience | | | | |  |
| Prism 10.1.2 | | Graphpad | | | | |  |
| Abbott Alinity hq analyzer | | Abbott | | | | |  |


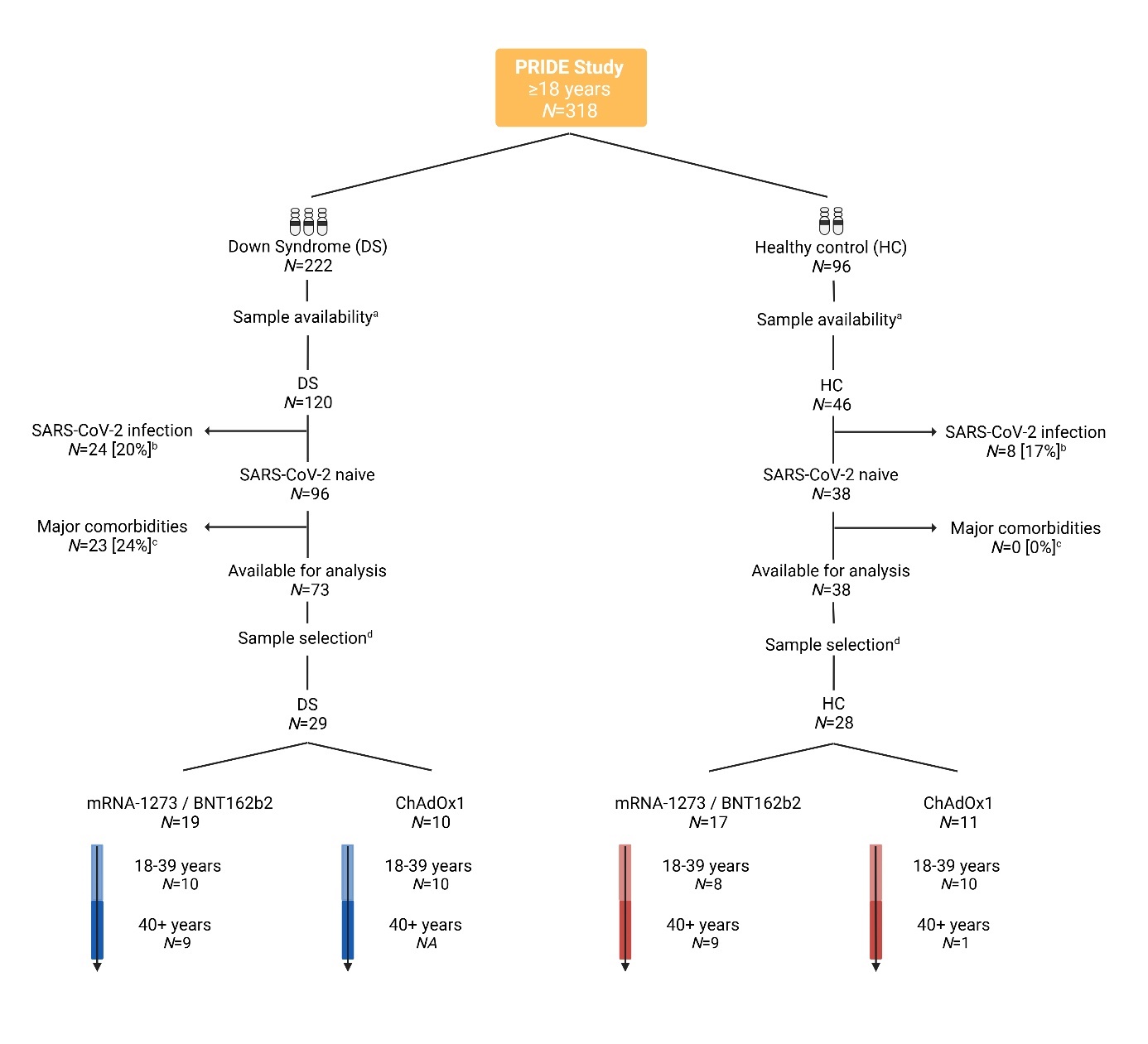


**Supplemental Figure 1: Flowchart of selected samples for AIM assay analysis at T3.**

^a^ participants were excluded in case of unknown or only one vaccination received, and in case of no PBMC availability at T3.

^b^ participants were excluded in case of a natural SARS-CoV-2 infection at T1, T2 or T3 as described in the methods.

^c^ participants were excluded in case of major comorbidities (primary or secondary immunodeficiency, congenital heart disease for which surgery was performed and Crohn’s disease) and in case of (immunosuppressive) medication such as systemic glucocorticoids or methotrexate and daily usage of antibiotics.

^d^ we selected approximately 10 participants per vaccine (mRNA vs vector) and age group (<40 vs ≥40 years), if available. We matched age and sex for DS and HC participants to the best of our capabilities.

Created in BioRender. Hensen, L. (2025) <https://BioRender.com/0zsvdy1>

**
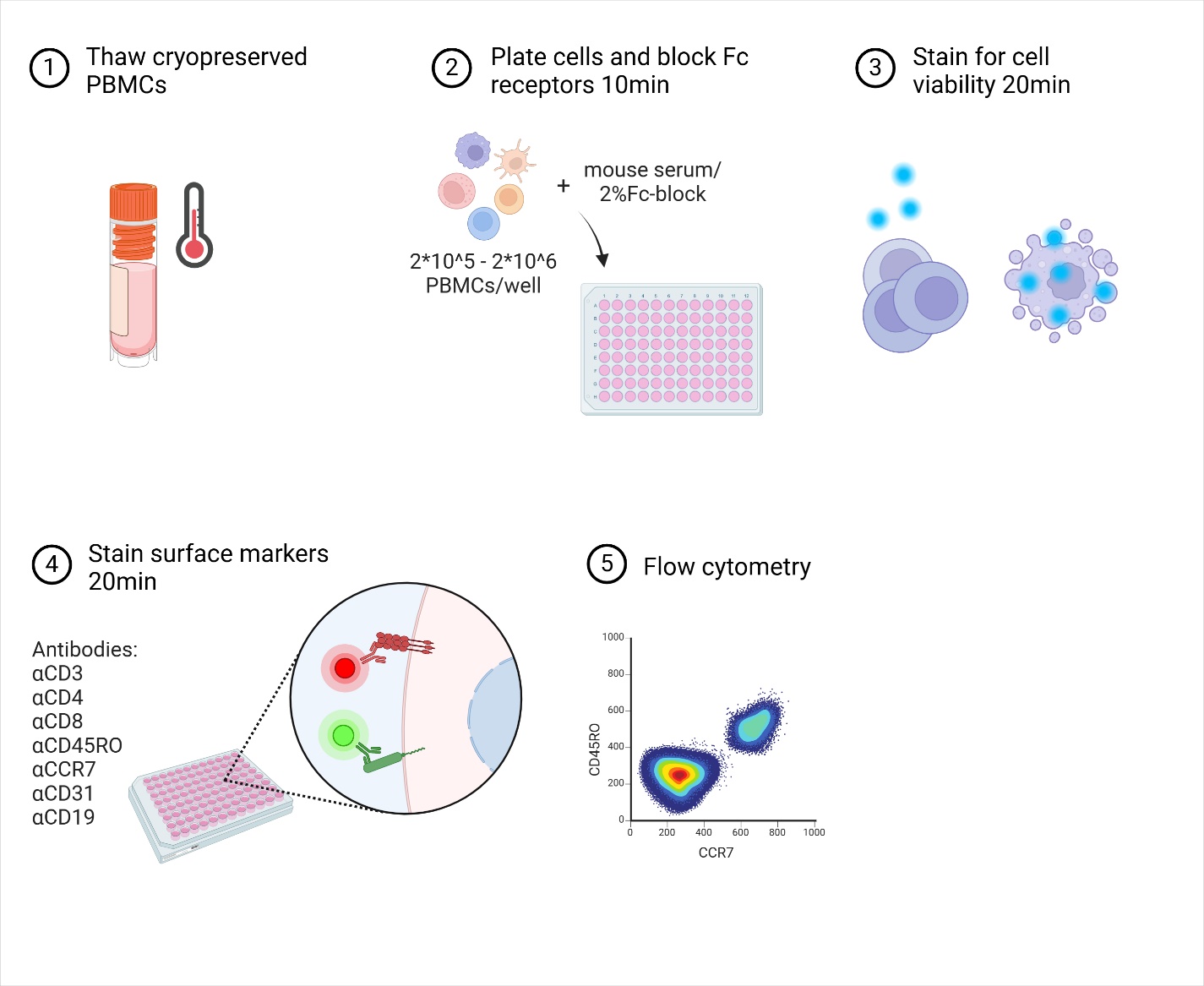
**

**Supplemental Figure 2: Flow cytometry protocol.** Cryopreserved cells were thawed in 15 mL RPMI1640 completed with 2% fetal calf serum, 100 U/ml penicillin, 100 μg/ml streptomycin, and 2 mM glutamine and washed twice. PBMCs were resuspended in RPMI1640 completed with 10% human AB serum, 100 U/ml penicillin, 100 μg/ml streptomycin, and 2 mM glutamine. Fc receptors of 2*10^5-2x10^6 PBMCs were blocked with normal mouse serum for 10 min at 4°C before cells were stained with a fixable viability dye for 20 min at 4°C. Fc receptors on PBMCs stained for B cells were additionally blocked with 2% Fc block for 10 min at 4°C. PBMCs were surface stained with antibodies against CD3, CD4, CD8, CCR7, CD45RO, and CD31 to phenotype T cells and CD19 to identify B cells in the presence of brilliant stain buffer for 20 min at 4°C.

Created in BioRender. Hensen, L. (2025) <https://BioRender.com/fopdu7n>


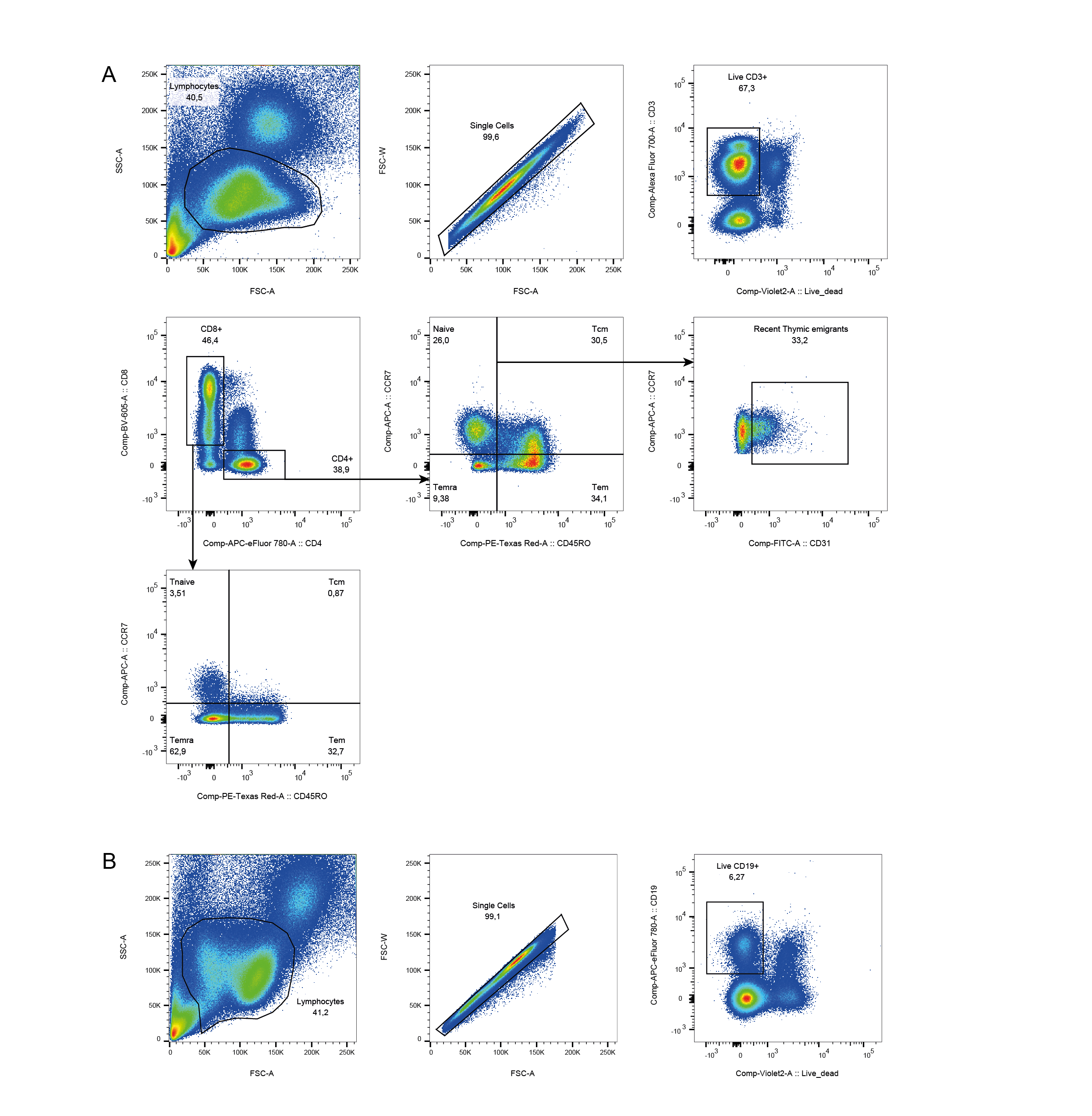


**
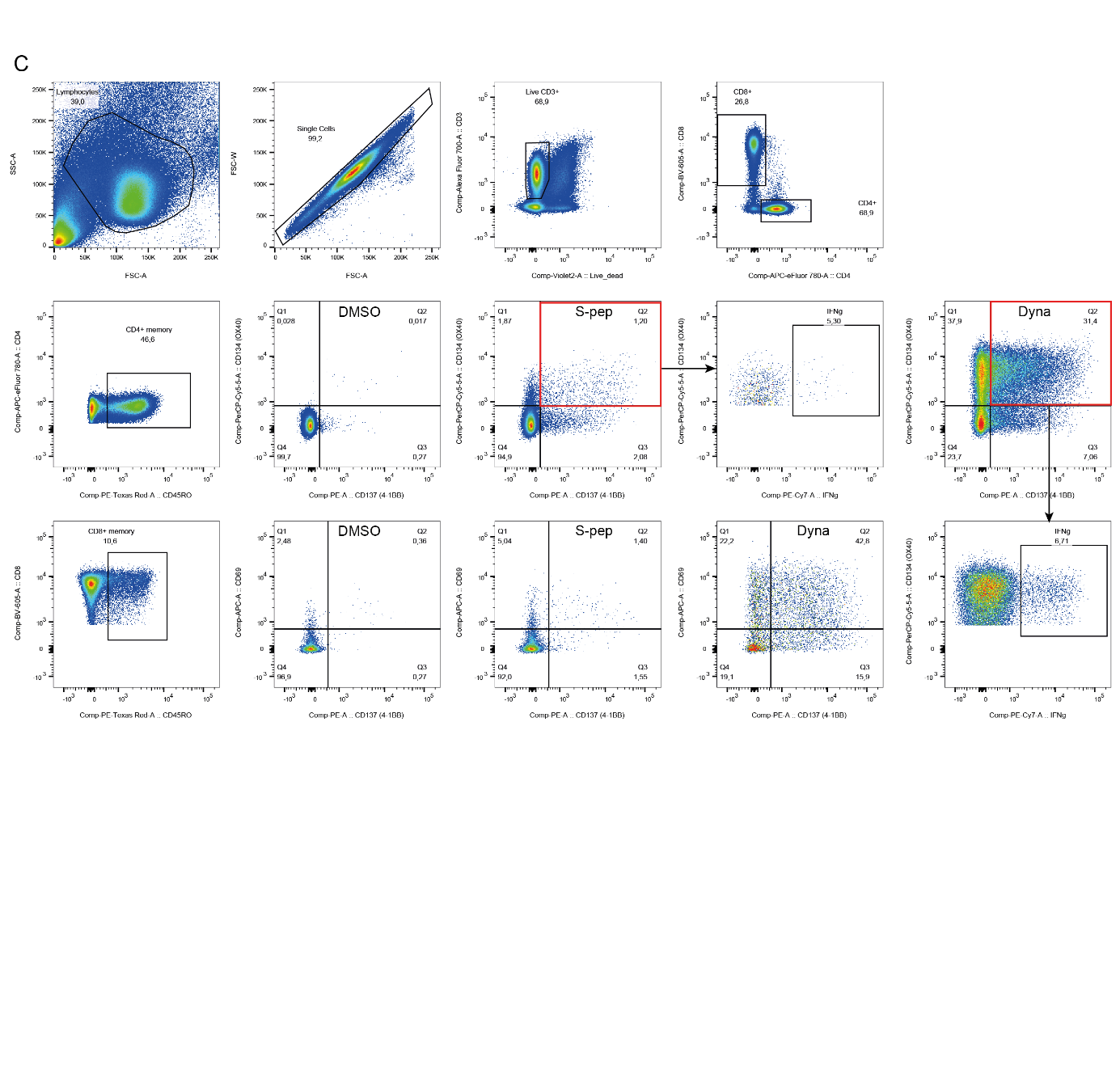
Supplemental Figure 3: FlowJo gating strategies.** (**A**) Gating strategy for T cells. Lymphocytes were gated for single, live CD3+ cells. CD3+ T cells were subdivided into CD3+CD4+ T helper cells and CD3+CD8+ T-cytotoxic cells. Naive and memory T cells were identified based on CCR7+ and CD45RO+ within CD4+ or CD8+ subsets. Naive CD4+ cells were gated for recent thymic emigrants (RTE) based on CD31+. (**B**) Gating strategy for B cells. Lymphocytes were gated for single, live CD19+ B cells. (**C**) Gating strategy of T cells after stimulation with DMSO, spike peptides or dynabeads. Single cells were gated for live CD3+ T cells and subdivided into CD3+CD4+ T helper cells and CD3+CD8+ T-cytotoxic cells. Memory T cells were identified by gating CD45RO+ within CD4+ or CD8+ subsets. SARS-CoV-2-specific memory T cells (AIM^+^ T cells) were defined as CD134+CD137+ for T helper cells and CD69+CD137+ for cytotoxic T cells. The DMSO-stimulated sample was used to set the cutoff gate for activation markers. AIM^+^ T cells were subgated for IFNγ production.


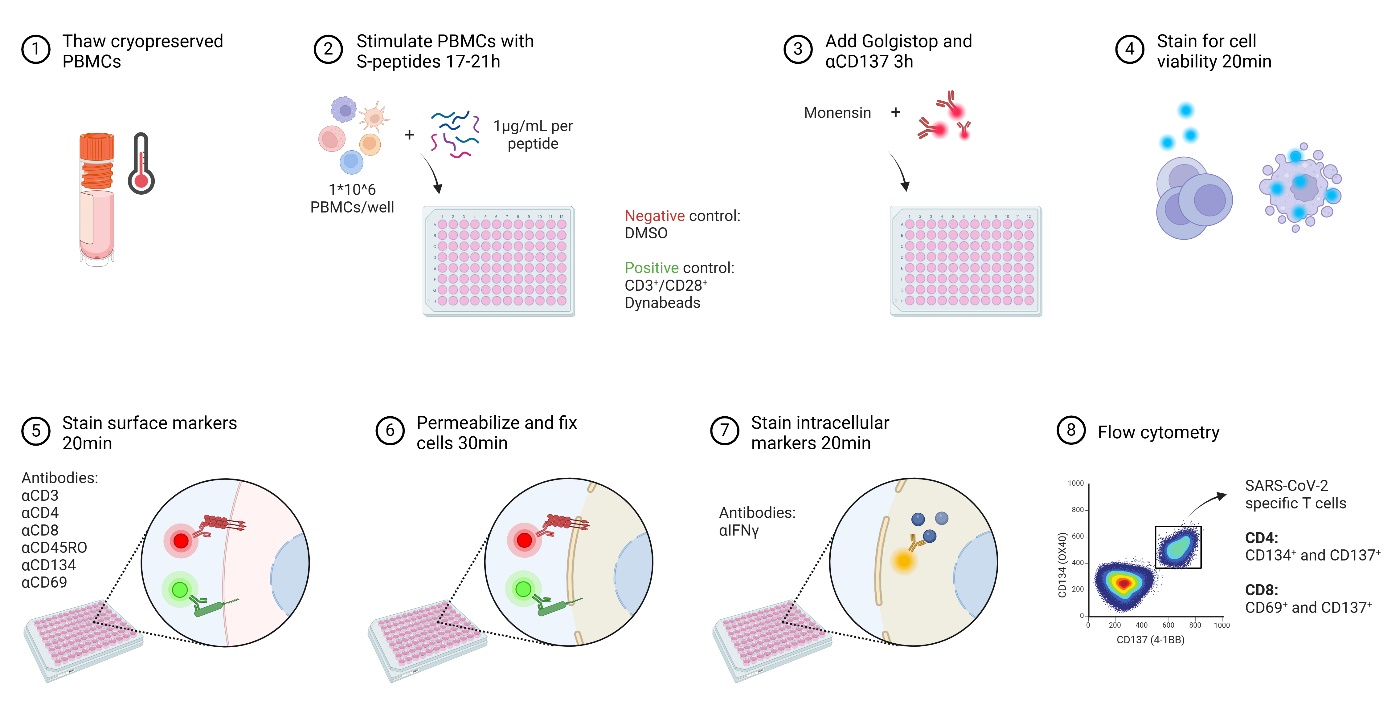
 **Supplemental Figure 4: AIM assay protocol.** Cryopreserved cells were thawed in 15 mL RPMI1640 completed with 2% fetal calf serum, 100 U/ml penicillin, 100 μg/ml streptomycin, and 2 mM glutamine and washed twice. PBMCs were resuspended in RPMI1640 completed with 10% human AB serum, 100 U/ml penicillin, 100 μg/ml streptomycin, and 2 mM glutamine (culture medium). 1x10^6 thawed PBMCs were stimulated for 20-24 hours at 37°C, 5% CO2 with a SARS-CoV-2 spike peptide pool (1 µg/mL per peptide) in 200 µL culture medium in 96-well U bottom plate. Cells were stimulated with an equimolar amount of DMSO (negative control), or with CD3/CD28 dynabeads (positive control). After 17-21 hours incubation, Golgistop (1500x diluted) and CD137 antibody were added for 3 hours in culture. Fc receptors were blocked with normal mouse serum for 10 min at 4°C before cells were stained with fixable viability dye for 20 min at 4°C. PBMCs were surface stained with antibodies against CD3, CD4, CD8, CD45RO, CD134, and CD69 in the presence of brilliant stain buffer for 20 min at 4°C. Finally, PBMCs were fixed and permeabilized with FoxP3 transcription factor staining buffer for 30 minutes at 4°C, and intracellular stained with anti-IFNγ for 20 min at 4°C.

Created in BioRender. Hensen, L. (2025) <https://BioRender.com/xuussq1>


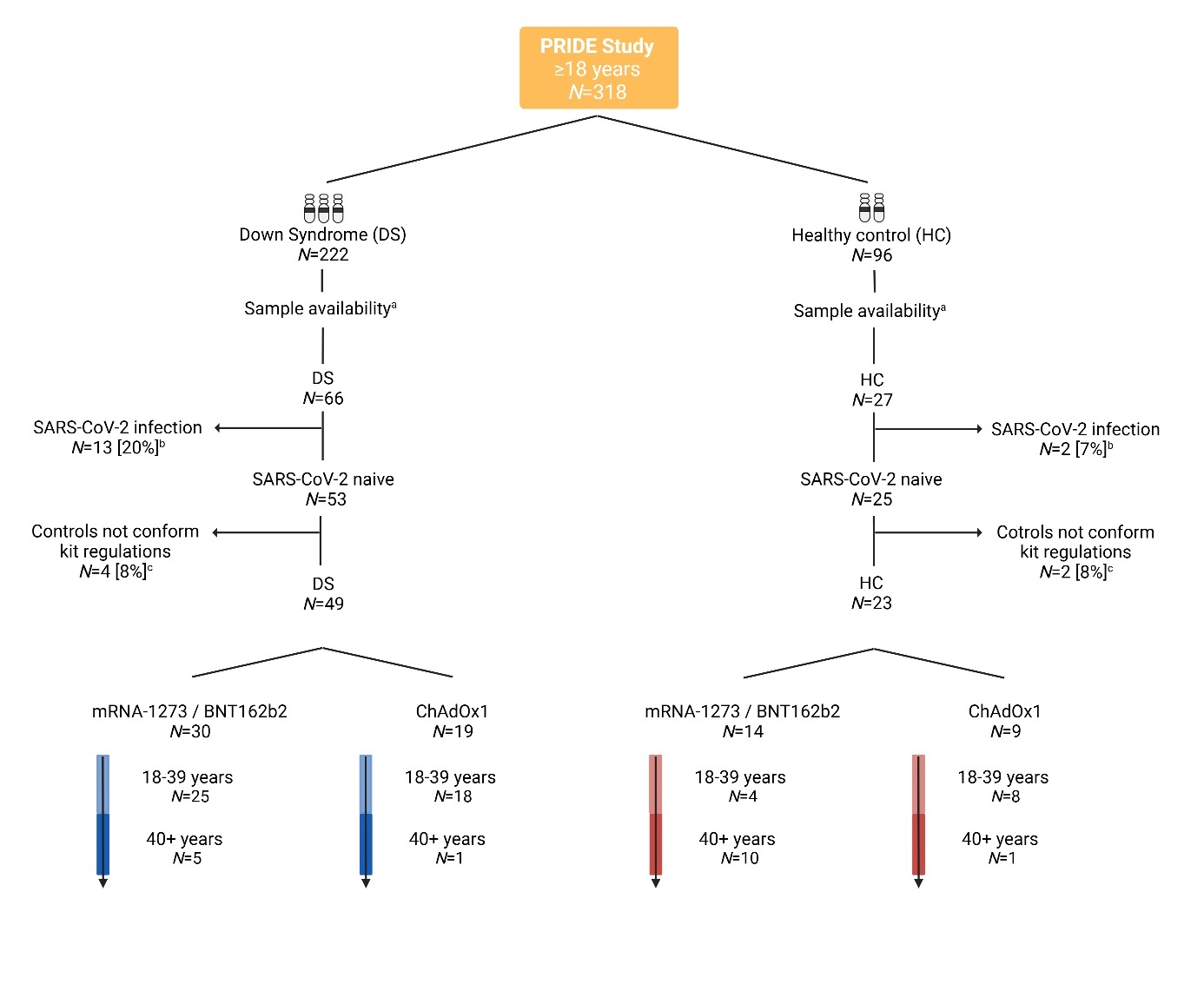


**Supplemental Figure 5: Flowchart of selected samples for IGRA analysis at T3.**

^a^ participants were excluded in case of unknown or only one vaccination received, and included based on fresh blood available for IGRA analysis at T3.

^b^ participants were excluded in case of a natural SARS-CoV-2 infection at T1, T2 or T3 as described in the methods.

^c^ participants were excluded from further analysis when the negative or positive control samples did not follow the regulations set by the manufacturer.

Figure created with Biorender.com.

Created in BioRender. Hensen, L. (2025) <https://BioRender.com/0zsvdy1>

**
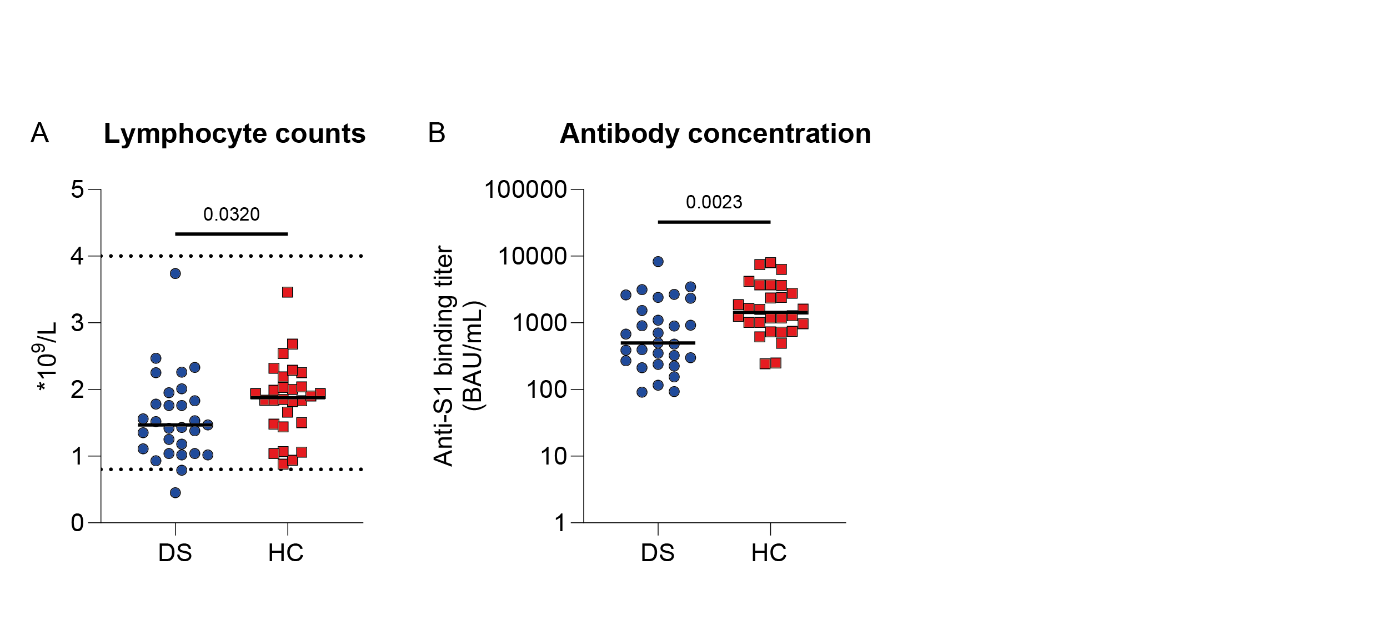
**

**Supplemental Figure 6: Baseline characteristics of the selected participants for AIM assay on T3.** (**A**) Absolute lymphocytes counts in DS and HC. Grey dotted lines indicate reference values for healthy adults. (**B**) Anti-S1 specific antibodies on T3, data obtained from (15). Significance in (**A**-**B**) was determined using Mann-Whitney tests and the median is shown as a black line. DS N=29 and HC N=28 for (**A-B**).

**
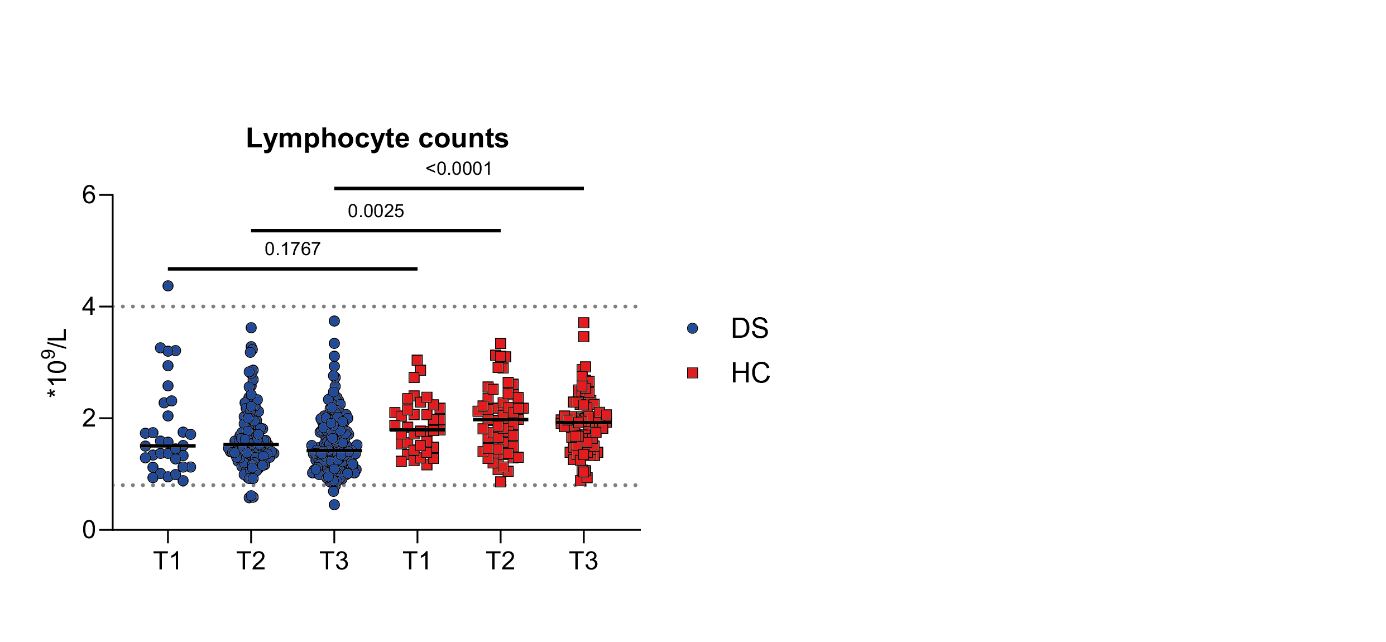
**

**Supplemental Figure 7: Lower absolute lymphocyte counts in DS**. Absolute lymphocyte counts for DS and HC at baseline (T1, DS N=32, HC N=37), after one (T2, DS N=114, HC N=54) and two SARS-CoV-2 vaccinations (T3, DS N=161, HC N=78). Grey dotted lines indicate reference values for healthy adults. Significance was determined using Kruskal-Wallis test and the mean is shown as a black line.

**
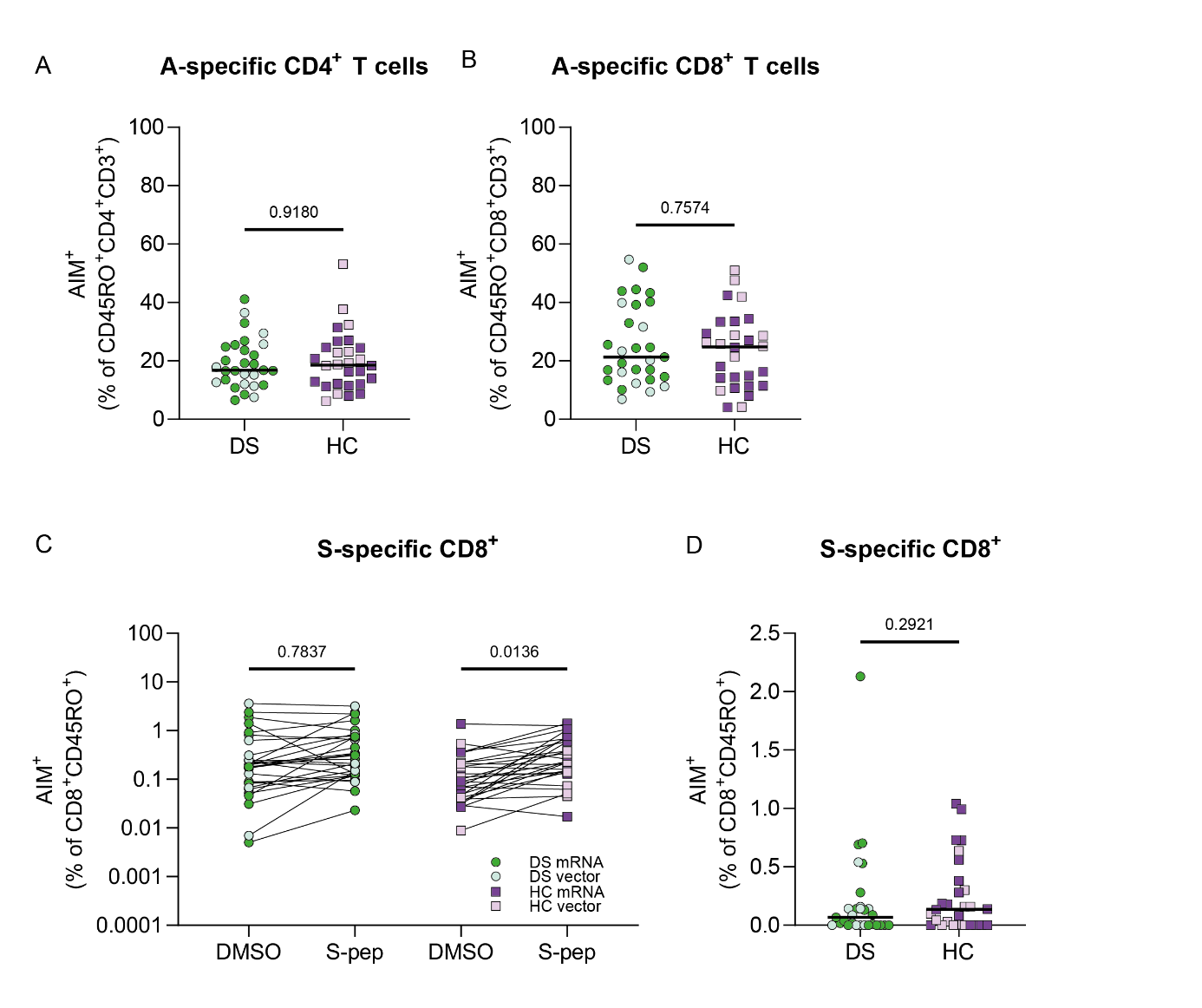
**

**Supplemental Figure 8: Detection of a-specific CD4^+^ and CD8^+^ T cells and S-specific CD8^+^ T cells by measuring upregulation of activation markers after two SARS-CoV-2 vaccinations.** (**A**) Percentage of AIM^+^ cells within the CD4^+^CD45RO^+^ and (**B**) CD8^+^CD45RO^+^ subsets after a-specific dynabeads stimulation. AIM^+^CD4^+^ cells are defined as CD134^+^CD137^+^ and AIM^+^CD8^+^ cells are defined as CD69^+^CD137^+^. (**C**) Percentage of AIM^+^ cells within the CD8^+^CD45RO^+^ subset after DMSO and spike peptide stimulation. (**D**) Percentage of AIM^+^ cells within the CD8^+^CD45RO^+^ subset after subtraction of the DMSO background. Significance in (**A, B** and **C**) was determined using Mann-Whitney tests and the median is shown as a black line. Significance in (**C**) was determined using the Kruskal-Wallis test. DS N=29 and HC N=28 for (**A-D**).


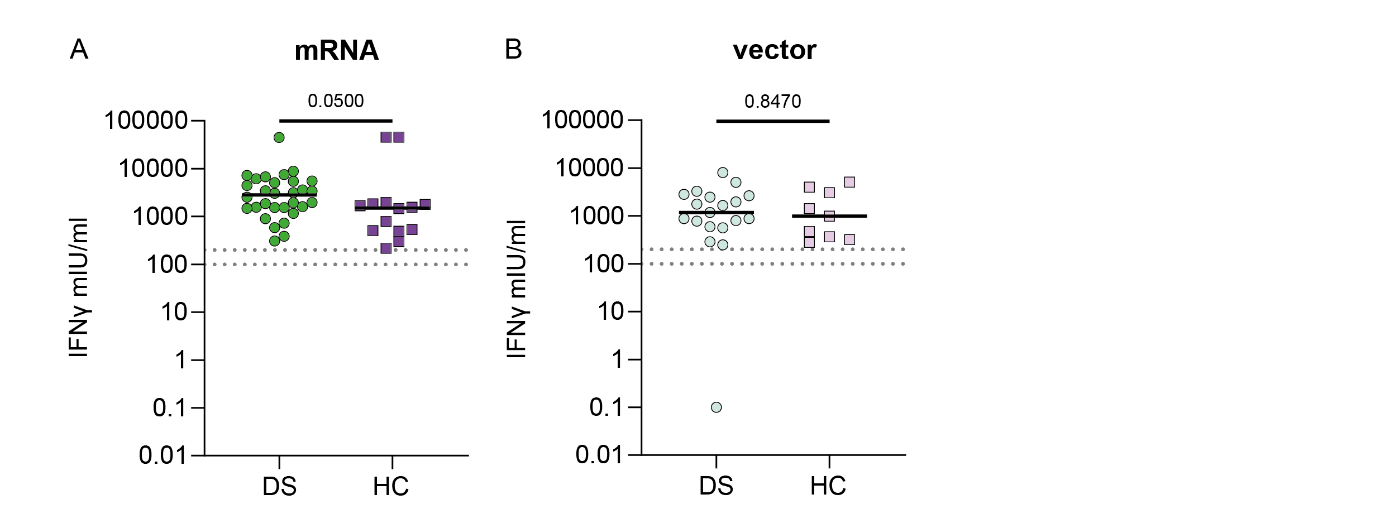


**Supplemental Figure 9: IFNγ production upon antigen re-stimulation at T3 after mRNA and vector vaccination.** (**A**) IFNγ (IU/ml) production by SARS-CoV-2-specific T cells at T3 after mRNA (DS N=30, HC N=14) or (**B**) vector (DS N=19, HC N=9) vaccination. An IFNγ concentration between 100-200 mIU/mL was considered a borderline vaccine reaction, and >200 mIU/mL a positive reaction, indicated by the dashed lines. Significance in (**A-B**) was determined using Mann-Whitney tests and the median is shown as a black line.

**Supplemental Table 2: Baseline characteristics T4 cohort AIM assay.**

|  | **DS (N=22)** | **HC (N=13)** | **p^a^** |
| --- | --- | --- | --- |
| Age (median, IQR^b^) | 28 (22-44) | 38 (27-53) | 0.0961 |
| Male gender (n, %) | 13 (59%) | 5 (38%) | 0.2380 |
| Ethnicity  North West European (n, %) | 20 (91%) | 13 (100%) | 0.5193 |
| Smoking (n, %) | 0 (0%) | 2 (15%) | 0.1311 |
| SARS-CoV-2 vaccine type primary series  BNT162b2 (n, %)  mRNA-1273 (n, %)  ChAdOx1 (n, %) | 10 (45%)  3 (14%)  9 (41%) | 6 (46%)  1 (8%)  6 (46%) | >0.9999 |
| Medical history  Thyroid disease (n, %)  Celiac disease (n, %)  Diabetes mellitus^c^ (n, %)  Congenital heart disease (n, %) | 8 (36%)  3 (14%)  2 (9%)  5 (23%) | 0 (0%)  0 (0%)  0 (0%)  0 (0%) | >0.9999 |

^a^Significance tested with Mann-Whitney U, Chi-square or Fisher’s exact test as described in the methods.

^b^IQR, interquartile range

^c^Including DM type 1 and type 2.

**Supplemental Table 3: Baseline characteristics T5 cohort AIM assay.**

|  | **DS (N=23)** | **HC (N=15)** | **p^a^** |
| --- | --- | --- | --- |
| Age (median, IQR^b^) | 28 (22-41) | 52 (31-59) | 0.0035 |
| Male gender (n, %) | 13 (59%) | 5 (33%) | 0.1617 |
| Ethnicity  North West European (n, %) | 21 (91%) | 14 (93%) | >0.9999 |
| Smoking (n, %) | 0 (0%) | 1 (7%) | 0.3947 |
| SARS-CoV-2 vaccine type primary series  BNT162b2 (n, %)  mRNA-1273 (n, %)  ChAdOx1 (n, %) | 10 (43%)  3 (13%)  10 (43%) | 8 (53%)  1 (7%)  6 (40%) | 0.8070 |
| Medical history  Thyroid disease (n, %)  Celiac disease (n, %)  Diabetes mellitus^c^ (n, %)  Congenital heart disease (n, %) | 9 (39%)  3 (13%)  2 (9%)  5 (22%) | 1 (7%)  0 (0%)  0 (0%)  0 (0%) | >0.9999 |

^a^Significance tested with Mann-Whitney U, Chi-square or Fisher’s exact test as described in the methods.

^b^IQR, interquartile range

^c^Including DM type 1 and type 2.


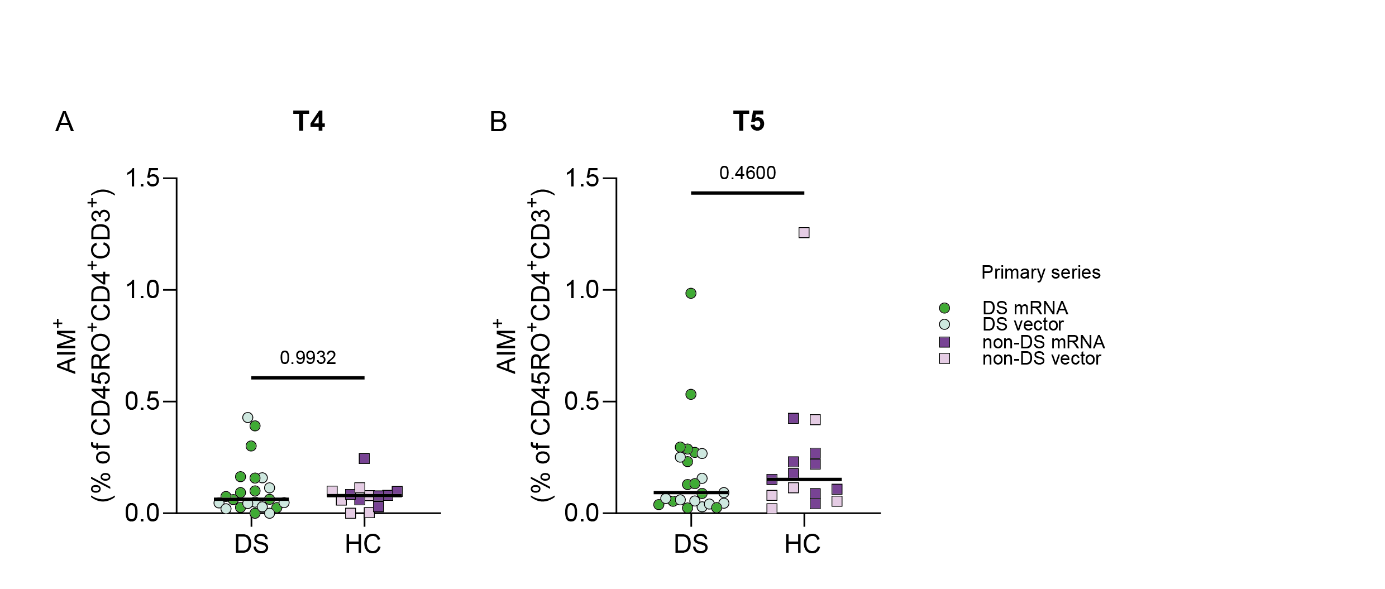


**Supplemental Figure 10: Similar percentage of SARS-CoV-2-specific CD4^+^ T cells in DS and HC before and after booster vaccination.** (**A**) Percentage of AIM^+^ cells within the CD4^+^CD45RO^+^ subset after subtraction of the DMSO background at T4 (DS N=22, HC N=13) and (**B**) T5 (DS N=22, HC N=15). AIM^+^CD4^+^ cells are defined as CD134^+^CD137^+^. Participants were grouped in the mRNA or vector group based on their primary SARS-CoV-2 vaccine regimen. Significance in (**A-B**) was determined using Mann-Whitney tests and the median is shown as a black line.


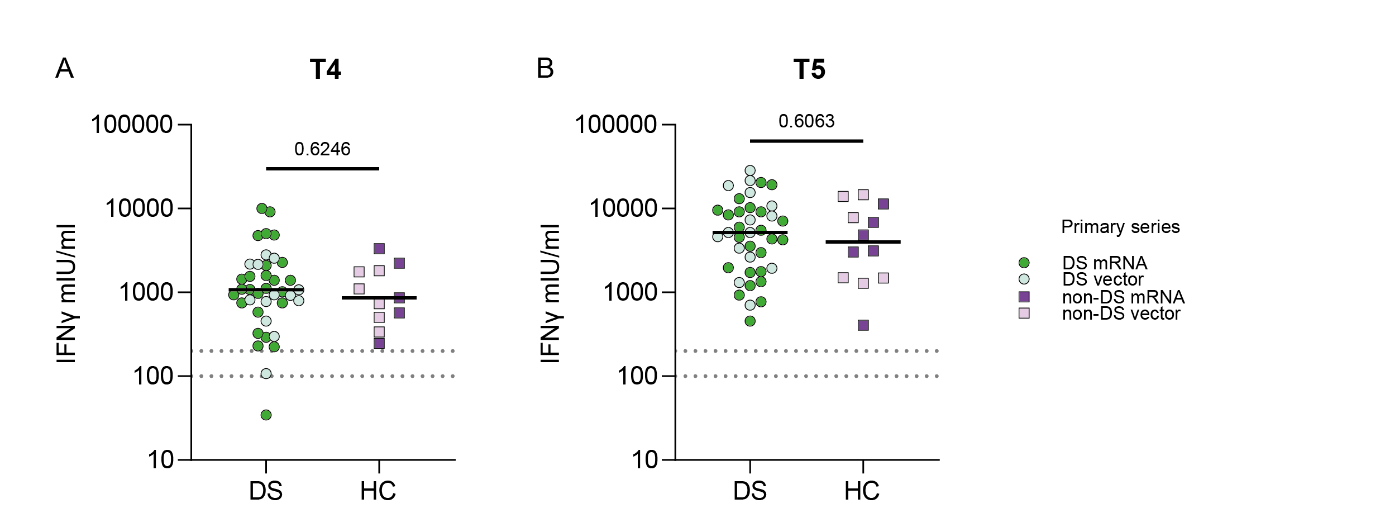


**Supplemental Figure 11: Comparable IFNγ production by SARS-CoV-2-specific T cells in DS and HC before and after booster vaccination.** (**A**) IFNγ (IU/ml) production by SARS-CoV-2-specific T cells in DS and HC before (DS N=39, HC N=11) and (**B**) after (DS N=39, HC N=12) booster vaccination. Participants were grouped in the mRNA or vector group based on their primary SARS-CoV-2 vaccine regimen. An IFNγ concentration between 100-200 mIU/mL was considered a borderline vaccine reaction, and >200 mIU/mL a positive reaction, indicated by the dashed lines. Significance in (**A-B**) was determined using Mann-Whitney tests and the median is shown as a black line.


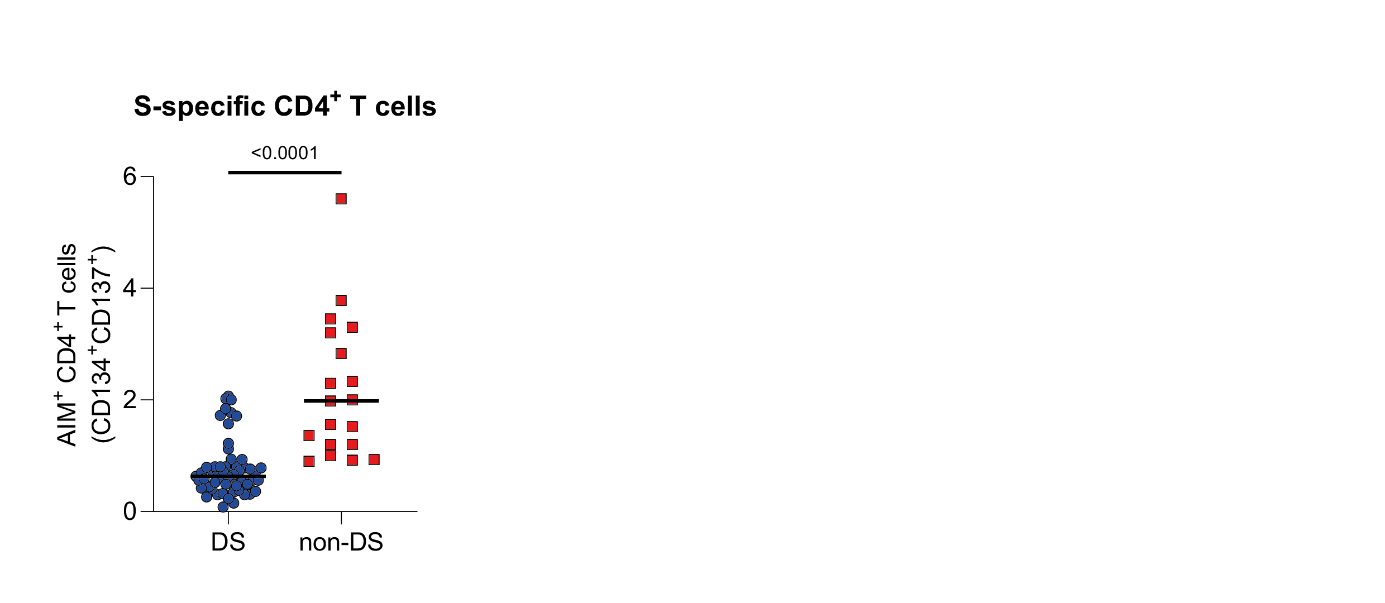


**Supplemental Figure 12: Re-analysis of data obtained from Esparcia-Pinedo et al.** **(21). Decreased SARS-CoV-2-specific CD4^+^ T cells in DS one to three months after two SARS-CoV-2 vaccinations.** Percentage of AIM^+^ cells within the CD4^+^ T cells after subtraction of the DMSO background in DS and HC. AIM^+^CD4^+^ T cells are defined as CD134^+^CD137^+^. Data was obtained from (21). Significance was determined using Mann-Whitney test and the median is shown as a black line.
